# Preliminary Evaluation of the Synergistic Antibacterial Effects of Selected Commercial Essential Oil Compounds Against Methicillin-Resistant *Staphylococcus aureus* ATCC 43300

**DOI:** 10.3390/antibiotics14070733

**Published:** 2025-07-21

**Authors:** Kacper Hartman, Maja Świerczyńska, Amelia Wieczorek, Piotr Baszuk, Iwona Wojciechowska-Koszko, Katarzyna Garbacz, Monika Sienkiewicz, Paweł Kwiatkowski

**Affiliations:** 1 Department of Diagnostic Immunology, Pomeranian Medical University in Szczecin, 70-111 Szczecin, Poland; kacperhartman@gmail.com (K.H.); majaswierczynska16@gmail.com (M.Ś.); wieczorekamelia03@gmail.com (A.W.); iwona.wojciechowska.koszko@pum.edu.pl (I.W.-K.); 2 Department of Genetics and Pathology, International Hereditary Cancer Center, Pomeranian Medical University in Szczecin, 71-252 Szczecin, Poland; piotr.baszuk@pum.edu.pl; 3 Department of Oral Microbiology, Medical Faculty, Medical University of Gdansk, 80-204 Gdansk, Poland; katarzyna.garbacz@gumed.edu.pl; 4 Department of Pharmaceutical Microbiology and Microbiological Diagnostics, Medical University of Lodz, 90-151 Lodz, Poland; monika.sienkiewicz@umed.lodz.pl

**Keywords:** essential oil compounds, antibacterial activity, checkerboard, synergism, MRSA

## Abstract

**Background/Objectives**: Growing antibiotic resistance is one of the most significant problems of current medicine. Various research efforts are focused on the search for new substances and their combinations as potential solutions to this problem. Essential oil compounds (EOCs) are considered promising candidates in this regard. However, the interactions between these natural compounds remain understudied. This study conducted a preliminary evaluation of the antimicrobial action of various commercial EOCs (1,8-cineole, eugenol, linalyl acetate, (-)-α-pinene, limonene, α-terpineol, DL-menthol, geraniol, farnesol, carvacrol, and myrcene) alone and in combination (*n =* 56) against methicillin-resistant *Staphylococcus aureus* strain (ATCC 43300). **Methods**: The following parameters were studied: antibacterial activity of EOCs alone and in combination using microdilution and checkerboard assays. **Results**: After the initial screening, geraniol, farnesol, linalyl acetate, carvacrol, (−)-α-pinene, α-terpineol, 1,8-cineole, and eugenol exhibited antibacterial activity against the tested strain and were, therefore, selected for further evaluation in the checkerboard assay. The checkerboard assay revealed 10 synergistic interactions, with farnesol demonstrating the highest number of synergistic combinations among the tested compounds. The results highlighted its high synergistic potential in combination with eugenol, linalyl acetate, (-)-α-pinene, α-terpineol, geraniol, and carvacrol. **Conclusions**: In conclusion, the results help elucidate the different interactions between EOCs and may be helpful in further applications of natural compounds as antimicrobial agents in wound dressings. Overall, the most promising compound was found to be farnesol.

## 1. Introduction

The global rise in antibiotic-resistant bacteria, such as methicillin-resistant *Staphylococcus aureus* (MRSA), is of growing concern to healthcare providers worldwide. Despite modern manufacturing methods, various organisations report that the development of new antibiotics is insufficient to combat this issue, prompting the urgent search for alternatives such as essential oils [EOs] and their compounds [EOCs] [1,2,3,4,5,6]. EOs are mixtures of various plant chemicals (mostly sesquiterpenes and monoterpenes); their volatility and resulting characteristic fragrance have historically defined them. Examples of EO-rich families include *Myrtaceae* and *Lamiaceae* [7,8]. It has been established that EOCs possess antimicrobial and antifungal properties, which likely stems from their primary role in plants to protect the host from infection and insect infestation [9]. EOCs are widely regarded as safe for consumption due to their widespread occurrence, with relatively uncommon adverse effects. However, the usage of these compounds has been hampered by their properties, such as volatility, insolubility in water, and oxidation potential [9,10,11]. These characteristics made them challenging to use in medical settings, and their medical applications were usually limited in scope, with studies typically conducted in vitro or on relatively small numbers of patients under limited conditions [3,12,13,14,15,16,17,18]. Thus, research on this topic often uses EOs directly derived from plants; however, the current study utilised their constituents in pure form. The reasoning behind this is as follows: EOs are known to vary in their composition [6,19,20], which could be a potential methodological flaw that would make the results difficult to replicate. Hence, this study emphasises the importance of using the purest form of compounds in their assessment to improve replicability.

*S. aureus* is a commonly occurring Gram-positive bacterium. Current statistics indicate that this bacterium may colonise up to 30% of the population, although most hosts exhibit no symptoms. [21]. *S. aureus* is an opportunistic pathogen, especially of primary concern to patients with compromised immunity. *S. aureus* can lead to infections in susceptible individuals, with possible complications including pneumonia, meningitis, infective endocarditis, osteomyelitis, and various inflammations of the skin, bones, joints, and soft tissues [21]. Among these, the MRSA strains have become one of the most significant challenges in clinical settings. The clinical classification of MRSA infections, in terms of the place of acquisition, is usually divided into hospital-acquired and community-acquired MRSA strains [22]. Due to the widespread use of various antibiotics in hospitals, MRSA strains have developed resistance to drugs such as methicillin, penicillin, oxacillin, cloxacillin, cefazolin, and cefoxitin. The resistance is the effect of producing penicillin-binding protein (PBP2a or PBP2′) that renders most of the semisynthetic penicillins ineffective. The PBP2a is encoded by the *mecA* gene located on a transposon cassette known as the staphylococcal cassette chromosome *mec* (*SCCmec*). This positioning of a mobile gene element (MGE) is crucial, as it enables the spread of methicillin resistance quickly among various strains [23].

Current forms of MRSA infection treatment typically employ a multidrug regimen to leverage various antimicrobial modes of action and prevent potential bacterial resistance. Typical treatment options usually include vancomycin in combination with daptomycin, ceftaroline, teicoplanin, telavancin, oxazolidinones, and linezolid [23,24]. However, all of these suffer from various side effects, such as nephrotoxicity (vancomycin, ceftroline, teicoplanin, televancin), hypokalemia (daptomycin), hearing and balance abnormalities (teicoplanin), or bone marrow abnormalities (linezolid, vancomycin, teicoplanin, telavancin) [25]. Hence, developing alternate treatment methods is of great clinical importance.

This study utilised 11 EOCs, namely geraniol, farnesol, linalyl acetate, carvacrol, (-)-α-pinene, α-terpineol, 1,8-cineole, eugenol, limonene, mircene, and menthol, due to their wide distribution in plant species and the extensive research regarding their antimicrobial properties [9,26]. The aforementioned EOCs are generally regarded as safe, which is a crucial advantage compared to traditional antibiotics, although there are singular reports of possible side effects, such as skin irritation, dermatitis, and ulcer formation [27].

Combinations of EOCs with antibiotics have been reported in the literature as a potential approach to addressing the growing problem of antibiotic resistance [28,29,30,31,32]. This approach can reduce the necessary doses of antibiotics and make it more challenging for bacteria to develop resistance due to the various mechanisms of action of antibiotics and EOCs. Despite evidence of synergistic effects between EOCs and antibiotics, a limited number of studies have assessed the direct combinatory effects of commercial EOCs without antibiotics against MRSA. Hence, this study took a different approach, avoiding the use of typical antibiotics. Instead, we aimed to evaluate the antibacterial efficacy of selected commercial EOCs—both individually and in combination—against the *S. aureus* ATCC 43300 (MRSA) strain, to determine whether these combinations can achieve antimicrobial effects with potential for practical and medical applications, such as in novel wound dressings. By singling out every important combination, the results of each assay are free from potentially unknown interactions between the different EOCs. Hence, this preliminary study evaluated the antibacterial activity of selected commercial EOCs, both alone and in combination, against the *S. aureus* ATCC 43300 (MRSA) strain.

## 2. Results

### 2.1. Evaluation of the MIC and MBC Values for EOCs Against MRSA Strain

Based on the performed study, the antibacterial activity of vancomycin hydrochloride (control) and eight of eleven EOCs (geraniol, farnesol, linalyl acetate, carvacrol, (-)-α-pinene, α-terpineol, 1,8-cineole, and eugenol) was confirmed against the MRSA strain. Antimicrobial activity was not observed in the case of menthol, myrcene, and limonene. Among all tested EOCs, geraniol exhibited the most potent antimicrobial effect (MIC = 6.82 ± 0.00 mg/mL; MBC = 6.82 ± 0.00 mg/mL), whereas (-)-α-pinene showed the weakest (MIC = 124.95 ± 0.00 mg/mL; MBC = 124.95 ± 0.00 mg/mL). It is worth emphasising that all the tested chemicals demonstrated bactericidal activity against the MRSA strain. Furthermore, it was also observed that the addition of Tween 80 (1%, *v*/*v*) or dimethyl sulfoxide (DMSO; 2%, *v*/*v*) had no impact on the growth of the MRSA strain. Detailed data on the obtained MIC and MBC values, the MBC/MIC ratio, and the bactericidal/bacteriostatic activity of the tested EOCs against the analysed strain are listed in Table 1.

### 2.2. Evaluation of the Antibacterial Activity of EOCs in Combination Against MRSA Strain

**Figure 1:**
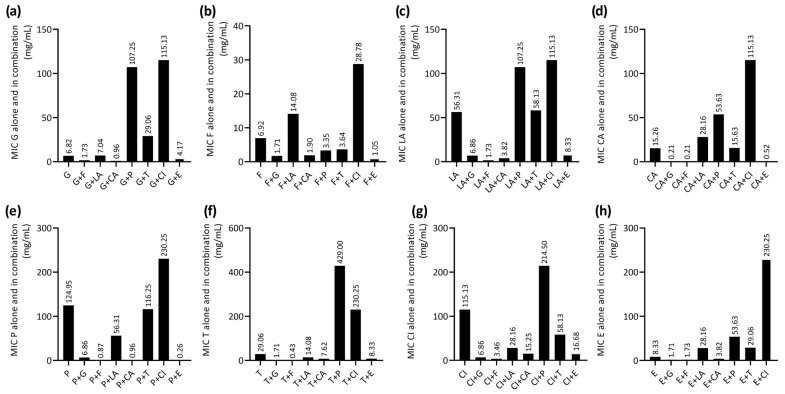
Minimum inhibitory concentrations (MICs) of essential oil compounds (EOCs) against methicillin-resistant *S. aureus* ATCC 43300 strain analysed alone and in combination. (**a**) G—geraniol; (**b**) F—farnesol; (**c**) LA—linalyl acetate; (**d**) CA—carvacrol; (**e**) P—(-)-α-pinene; (**f**) T—α-terpineol; (**g**) CI—1,8-cineole; (**h**) E—eugenol. Each bar represents the MIC value of a single EOC or a combination of two compounds (EOC + EOC).

Based on the interaction study of 28 combinations of EOCs, it was noted that half of them (geraniol–farnesol, geraniol–carvacrol, farnesol–linalyl acetate, farnesol–carvacrol, farnesol–(-)-α-pinene, farnesol–α-terpineol, farnesol–eugenol, carvacrol–(-)-α-pinene, carvacrol–eugenol, geraniol–eugenol, farnesol–1.8-cineole, linalyl acetate–carvacrol, and carvacrol–α-terpineol) showed a decrease in MIC values for each EOC. The lowest decrease in EOC MICs was observed for the following combinations: geraniol–carvacrol (geraniol—1/32 × MIC, carvacrol—1/16 × MIC), farnesol–(-)-α-pinene (farnesol—1/8 × MIC, (-)-α-pinene—1/36 × MIC), and farnesol–carvacrol (farnesol—1/32 × MIC, carvacrol—1/8 × MIC). Details of the assessment of the MIC values of the EOCs analysed in combination are shown in Figure 1.

**Figure 2:**
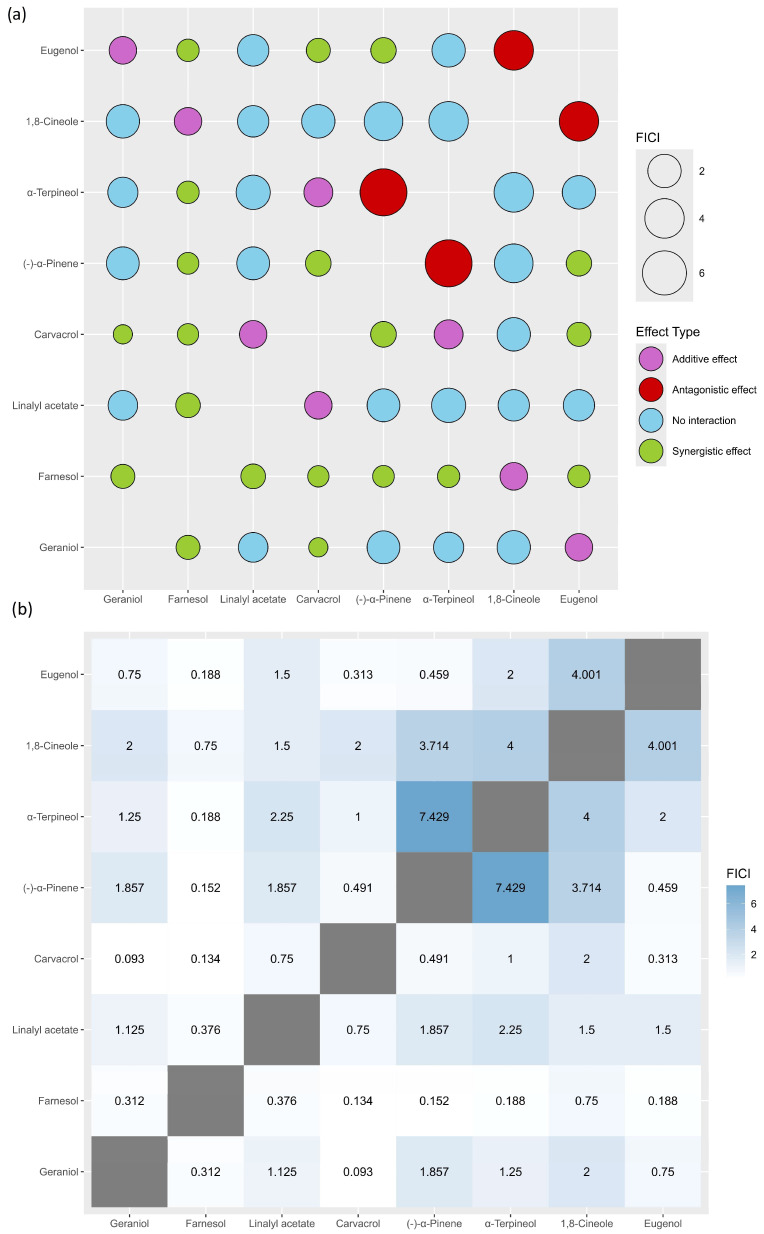
Bubble plots (**a**) and heatmap (**b**) showing the fractional inhibitory concentration index (FICI) values of essential oil compounds (EOCs) analysed in combination against methicillin-resistant *S. aureus* ATCC 43300 strain. FICI was calculated for each combination (for details, see Materials and Methods, Section 4.4), the values of which were used to define the type of interaction. FICI values were interpreted accordingly as synergistic effect (FICI < 0.5), additive effect (0.5 ≤ FICI ≤ 1.0), no interaction (1.1 < FICI ≤ 4.0), or antagonistic effect (FICI > 4.0).

The checkerboard assay revealed the following interactions of EOCs: 35.7% (synergistic effect), 14.3% (additive effect), 39.3% (no interaction), and 10.7% (antagonistic effect). It is worth noting that farnesol alone showed 60.0% synergistic effects. Nevertheless, the geraniol–carvacrol combination obtained the lowest FICI value (0.093). Details of the FICI values of the analysed EOCs are presented in Figure 2.

The Fractional Inhibitory Concentration Index (FICI) was calculated for each combination (for details, see Materials and Methods, Section 4.4), the values of which were used to define the type of interaction. FICI values were interpreted accordingly as synergistic effect (FICI < 0.5), additive effect (0.5 ≤ FICI ≤ 1.0), no interaction (1.1 < FICI ≤ 4.0), or antagonistic effect (FICI > 4.0).

## 3. Discussion

Growing antibiotic resistance poses a serious threat to public health systems worldwide [22]. The search for alternative therapies to antibiotics continues, and EOCs may be one of the possible solutions to mitigate this problem. There is a scarcity of research that details the combinations of EOCs against bacteria. Additionally, a considerable proportion of the studies used EOs instead of their constituents, which can lead to discrepancies in the reported MIC values. This study’s results help elucidate how EOCs can be utilised to create more efficient combinations. Through this screening, farnesol was shown to have the lowest absolute MIC values in its synergistic effects, leading to the highest number of synergisms. Farnesol is a sesquiterpene alcohol in many plants and is a component of lemongrass, cyclamen, rose, citronella, and neroli EOs [33]. Its pyrophosphate precursor forms the base for almost all other sesquiterpenes [34]. It exhibits various pharmacological properties, including antimicrobial, antitumour, and antioxidant activities, and may be beneficial in the prevention and treatment of different diseases [35]. This shared pathway might explain the overwhelmingly synergistic results of farnesol, and its structure may not interfere with the activity or biosynthesis pathways of other EOCs. Moreover, Kaneko et al. demonstrated that farnesol inhibits the mevalonate pathway in *S. aureus*, thereby adding a mode of action beyond cell membrane disruption [36]. It is worth emphasising that much of literature data reported synergistic effects between farnesol and antibiotics against bacteria (for example, *Staphylococcus* spp., *Enterococcus faecalis*) [37]. Regarding MRSA, a study by Kuroda et al. reported strong synergistic effects of farnesol with ampicillin, oxacillin, and cefoxitin [38].

Carvacrol, the main constituent of oregano and thyme EOs, is a promising synergistic agent. The best synergistic effect in this study was observed with geraniol (FICI = 0.09), which is considered an important result that may be useful for further tests, as these compounds do not usually occur in the same plants (geraniol is, for example, in *Geranium* spp., *Cymbopogon* spp., whereas carvacrol is in *Origanum* spp. and *Thymus* spp.) [39,40]. Carvacrol has been widely studied against pathogenic bacteria, such as *S. aureus* and *Bacillus cereus* [41]. Its utility is evidenced by its ability to inhibit Gram-negative bacteria that are usually more resilient to EOCs [9]. Carvacrol exhibits a diverse mode of action, with its primary effect on bacterial membrane permeability; however, inhibition of specific proteins, such as GroEL and flagellin, may also play a role [9]. Numerous studies in the available literature confirm its synergistic effect with antibiotics such as cefixime, amoxicillin, ciprofloxacin, erythromycin, meropenem, gentamicin, and miconazole, especially against *S. aureus* and group A streptococci [29,42,43,44]. Hence, the current study contributes to the body of evidence that carvacrol can be an efficient antimicrobial agent, with a possible synergistic effect when combined with EOCs, such as geraniol (FICI = 0.093), farnesol (FICI = 0.13), linalyl acetate (FICI = 0.75), and eugenol (FICI = 0.31).

The current study also indicated the antistaphylococcal potential of eugenol. This compound reached a low MIC value (MIC = 8.33 mg/mL). This result concurs with previous studies that have shown the antibacterial effects of eugenol against *S. aureus*, *Escherichia coli*, and *Salmonella typhimurium* [9,26,45], as well as with the MIC values of eugenol from our previous experiments [46]. Its mode of action is linked to free hydroxyl groups, which lead to the destabilisation of bacterial cell membranes and the inhibition of bacterial proteins, such as adenosine triphosphatase, amylases, or proteases [9]. Based on the results of the current study, it was demonstrated that it could synergise with farnesol (FICI = 0.19), carvacrol (FICI = 0.31), and (-)-α-pinene (FICI = 0.46). Synergisms of eugenol were noted in other studies that showed the combinations of eugenol with cinnamaldehyde (against *E. coli*), linalool (against *Listeria monocytogenes*, *Klebsiella aerogenes*, *Pseudomonas aeruginosa*, and *E. coli*), and menthol (against *L. monocytogenes*, *E. aerogenes*, and *P. aeruginosa*) [26]. Additionally, some studies showed that combining antibiotics with eugenol against MRSA is inefficient [26]. The conflicting results suggest the need for further studies to accurately describe the mode of action of eugenol, both alone and in combination with other compounds.

(-)-α-Pinene is typically regarded as an EOC with weak antibacterial activity alone, which was also confirmed in this study [9,47]. Its results on MRSA showed low antimicrobial activity, and its mode of action is possibly related to the activation of heat shock proteins [48,49]—studies combining (-)-α-pinene with antibiotics revealed mainly indifferent results [49]. The results generally concur with the observations made in this study, as synergism was observed only with farnesol. Notably, (-)-α-pinene can be produced from geraniol in acidic solutions; hence, the study stresses the importance of chemical modifications on the synergistic properties of essential oil compounds [50].

Geraniol has been studied against various bacteria (including *S. aureus*) and has shown synergistic effects when combined with chloramphenicol, norfloxacin, and tetracycline [40]. It is worth noting that studies have shown antagonistic interactions of geraniol with tetracyclines against bacteria such as *K. pneumoniae*, *P. aeruginosa*, and *S. aureus* [51]. Regarding MRSA, it has been demonstrated that geraniol can effectively reduce biofilm formation and increase the therapeutic potential of vancomycin [52]. Its antimicrobial action is linked to its ability to alter cell membrane permeability [40]. In the checkerboard assay conducted in this study, geraniol exhibited strong synergistic effects, particularly with farnesol and carvacrol. Since both are terpenoids, it is possible that combining these compounds may lead to strong synergistic effects, warranting further study.

Linalyl acetate is also a terpenoid, primarily found in *Lavandula* species. It exhibits weak antimicrobial activity but has been effectively used in combination with beta-lactam antibiotics against MRSA strains [31]. Nevertheless, in the current study, aside from farnesol, linalyl acetate did not demonstrate any synergistic effects. However, its combinations with carvacrol and eugenol, which were classified as additive effects (FICI = 0.75), also have potential utility. However, linalyl acetate remains understudied in this aspect.

1,8-Cineole (eucalyptol) exhibits weak antimicrobial properties against MRSA [30,46]; however, it has been shown to induce oxidative stress in MRSA, which can lead to the reversal of antibiotic resistance [53]. 1,8-cineole was demonstrated to have the least promising interactions with the studied compounds. This observation may prove valuable in future applications, as it may be necessary to exclude it from products containing multiple EOCs or, at the very least, use it separately before using other EO-based products. Aside from indifference, 1,8-cineole showed two antagonisms with α-terpineol and (-)-α-pinene. A possible explanation may be that eucalyptol usually is the main component of its natural oil (eucalyptus EO). However, it is worth noting that (-)-α-pinene exists in low concentrations in some of the eucalyptus EOs [54].

The results also showed interesting effects of (-)-α-pinene and α-terpineol. The two belong to the same class of compounds, namely terpenoids [9], both being considered weakly antimicrobial (for example, *S. aureus*, *Streptococcus pneumoniae*, and *Streptococcus pyogenes* in the case of (-)-α-pinene [48] and *S. typhimurium*, *L. monocytogenes*, and *S. aureus* in the case of α-terpineol [1]) on their own [9,47]. However, some studies have shown their potential for synergism, namely by increasing cell membrane penetration [1,2]. The current study revealed a synergistic effect between α-terpineol and farnesol (FICI = 0.19). Other studies have shown that α-terpineol can strongly synergise with terpinene-4-ol against *S. aureus* (including MRSA), *E. coli*, and *P. aeruginosa* [55], as well as exhibit synergism with gentamicin against *P. aeruginosa* [56]. Moreover, α-terpineol showed intense antagonisms in combination with (-)-α-pinene. The exact mechanism remains unknown, but it is possible that they either compete in their mode of action or interact with each other in some way. If further studies confirm this interaction, it would be highly advisable to avoid combining the two chemicals.

## 4. Materials and Methods

### 4.1. Reference Strain and Growth Conditions

In this study, a single reference strain was used, *S. aureus* ATCC 43300, belonging to the Chair of Microbiology, Immunology and Laboratory Medicine, Pomeranian Medical University in Szczecin (Poland) collection. This strain is a well-characterised and widely accepted methicillin-resistant strain used in antimicrobial susceptibility testing. This choice allowed for standardised comparisons across essential oil compounds and minimised variability that may arise from clinical isolates with unknown or inconsistent resistance profiles. Before each study stage, the strain was cultured on Columbia agar supplemented with 5% sheep blood (bioMérieux, Warsaw, Poland) and incubated for 18 h at 37 °C under aerobic conditions.

### 4.2. Chemicals and Preparation of Dilution

Eleven EOCs were used in this study: 1,8-cineole (99% purity; lot number BCCG5541; Sigma-Aldrich, Darmstadt, Germany), eugenol (≥98% purity; lot number A0439846; Sigma-Aldrich, Darmstadt, Germany), linalyl acetate (≥97% purity; lot number SHBM3367; Sigma-Aldrich, Darmstadt, Germany), (-)-α-pinene (≥97% purity; lot number 584LEX; Pol-Aura, Zawroty, Poland), limonene (≥99% purity; lot number MKCN4494; Sigma-Aldrich, Darmstadt, Germany), α-terpineol (98% purity; lot number 467PBZ; Pol-Aura, Zawroty, Poland), DL-menthol (>99% purity; lot number 734ADL; Pol-Aura, Zawroty, Poland), geraniol (>95% purity; lot number 898GUK; Pol-Aura, Zawroty, Poland), farnesol (95% purity; lot number SHBM7150; Sigma-Aldrich, Darmstadt, Germany), carvacrol (≥98% purity; lot number 815GKV; Pol-Aura, Zawroty, Poland), and myrcene (90% purity; lot number KBC856 Pol-Aura, Zawroty, Poland).

Each EOC was diluted in an appropriate detergent for testing antibacterial activity. For 1,8-cineole, eugenol, linalyl acetate, (-)-α-pinene, limonene, geraniol, farnesol, carvacrol, and myrcene, Tween 80 (Sigma-Aldrich, Darmstadt, Germany) was used at a concentration not exceeding 1% (*v*/*v*). In contrast, for α-terpineol and DL-menthol, DMSO (Loba Chemie, Mumbai, India) was used at a concentration not exceeding 2% (*v*/*v*).

The control was vancomycin hydrochloride (MIP Pharma Poland, Gdansk, Poland).

### 4.3. Evaluation of MIC, MBC, MBC/MIC Ratio, and the Activity of Chemicals Against Reference Strain

MICs of EOCs against the reference strain were determined using the broth microdilution method in a 96-well microplate, following the guidelines of the Clinical and Laboratory Standards Institute (CLSI) [27]. Initially, the EOCs were diluted in Tween 80 or DMSO and then further diluted in Mueller–Hinton broth (MHB; Sigma-Aldrich, Darmstadt, Germany) to obtain the appropriate testing concentrations (500–0.01 µL/mL). Subsequently, 50 µL of each EOC dilution and 50 µL of staphylococcal suspension at 10^6^ colony-forming units/mL (CFU/mL) were added to each well. After incubation for 18 h at 37 °C, MICs were evaluated using resazurin sodium salt (Sigma-Aldrich, Darmstadt, Germany) [57]. The lowest concentration of the EOC at which the well remained blue was considered the MIC. The color change was measured visually. Knowing the density of all EOCs, the final results were expressed in milligrams per milliliter (mg/mL). Vancomycin hydrochloride (used as a control) was first diluted in sterile water and then further diluted in MHB to achieve the appropriate concentrations (4081–0.0005 mg/mL).

MBCs were determined by transferring 20 µL of the post-culture medium at concentrations ≥ MIC into a 96-well microplate. Each well was then filled with 100 µL of fresh Mueller–Hinton broth and incubated at 37 °C for 18 h. The lowest concentration of the EOC at which visible bacterial growth was observed in the well was considered the MBC [31]. To assess the activity of EOCs, the MBC/MIC ratio was calculated, with ratios ≤ 4 and >4 indicating bactericidal and bacteriostatic effects, respectively [58].

### 4.4. The Checkerboard Assay

To determine interactions between selected EOCs, a checkerboard method was employed, following the previously described methodology [59]. Each well contained a total volume of 50 μL, consisting of 25 μL of each respective EOC (diluted in Tween 80 or DMSO), prepared in serial dilutions in MHB according to their decreasing concentrations. Subsequently, 50 μL of MHB containing the staphylococcal suspension (10^6^ CFU/mL) was added to each well. After 18 h of incubation at 37 °C, the MIC for the combinations was assessed by resazurin sodium salt (0.02%) [57]. Knowing the density of all EOCs, the final results were expressed in milligrams per milliliter (mg/mL). A well containing 100 μL of broth served as the negative control, while a well with 50 μL of broth and 50 μL of staphylococcal suspension was used as the positive control.

For each combination, a fractional inhibitory concentration (FIC) and fractional inhibitory concentration index (FICI) were established using the following formulas:

$$FIC=\frac{MIC\;of\;EOC\;in\;combination}{MIC\;of\;EOC\;alone}$$

FICI=FIC of EOC1+FIC of EOC2

Results were interpreted as follows: synergistic effect (FICI < 0.5), additive effect (0.5 ≤ FICI ≤ 1.0), no interaction (1.1 < FICI ≤ 4.0), or antagonistic effect (FICI > 4.0).

### 4.5. Statistical Analysis

MIC/MBC results are expressed as the mean ± standard deviation from three measurements. All data management, calculations, and graphics were performed using the R statistical environment 4.5.0 (Vienna, Austria) [60], incorporating additional packages: ggplot2 (3.5.2) [61], reshape2 (1.4.4) [62], and svglite (2.1.3) [63].

## 5. Conclusions

This interaction study demonstrated that out of the 28 tested EOC combinations against *S. aureus* ATCC 43300 (MRSA), 10 exhibited synergistic effects, 4 were additive, 11 showed indifference, and 3 displayed antagonistic effects. Among all the EOCs combinations analysed, farnesol showed the highest number of synergistic interactions, confirming its high antibacterial potential in combination with other EOCs (eugenol, linalyl acetate, (-)-α-pinene, α-terpineol, geraniol, farnesol, carvacrol), excluding 1,8-cineole. While the current study provides valuable preliminary insights into the synergistic antibacterial potential of selected EOCs, future investigations involving larger sample sizes and more rigorous statistical evaluations will be essential to validate and expand upon these findings.

The natural compounds investigated in this study—particularly farnesol, geraniol, carvacrol, and eugenol—show significant antimicrobial potential against MRSA, primarily when used in well-chosen combinations. It provides a foundation for further in-depth analyses to evaluate the therapeutic applicability of these combinations in treating infections caused by multidrug-resistant strains. Future studies should focus on confirming the activity of such mixtures under preclinical and clinical conditions, determining optimal application methods, and elucidating the molecular basis of their synergistic effects. A significant limitation is the use of only one strain; therefore, future studies should consider using clinical strains to evaluate future clinical applications properly. Only a comprehensive research approach will allow for a balanced assessment of the true potential of these compounds and help determine whether they could serve as an effective complement or alternative to conventional antibiotic therapy in the future.

## Figures and Tables

**Table 1:** Minimum inhibitory concentration (MIC) values, minimum bactericidal concentration (MBC) values, MBC/MIC ratio, and the bactericidal/bacteriostatic activity of the essential oil compounds (EOCs) against methicillin-resistant *S. aureus* ATCC 43300 strain.

EOC	MIC (mg/mL)	MBC (mg/mL)	MBC/MIC Ratio	Activity
Geraniol	6.82 ± 0.00	6.82 ± 0.00	1	Bactericidal
Farnesol	6.92 ± 0.00	18.46 ± 0.00	3	Bactericidal
Linalyl acetate	56.31 ± 0.00	225.25 ± 0.00	4	Bactericidal
Carvacrol	15.26 ± 0.00	30.5 ± 0.00	2	Bactericidal
(-)-α-Pinene	124.95 ± 0.00	124.95 ± 0.00	1	Bactericidal
α-Terpineol	29.06 ± 0.00	29.06 ± 0.00	1	Bactericidal
1,8-Cineole	115.13 ± 0.00	115.13 ± 0.00	1	Bactericidal
Eugenol	8.33 ± 0.00	8.33 ± 0.00	1	Bactericidal
Vancomycin hydrochloride (control)	0.0005 ± 0.0000	0.0005 ± 0.0000	1	Bactericidal

## Data Availability

Data are contained within the article.
